# Combining Neuropsychological Assessment with Neuroimaging to Distinguish Early-Stage Alzheimer’s Disease from Frontotemporal Lobar Degeneration in Non-Western Tonal Native Language-Speaking Individuals Living in Taiwan: A Case Series

**DOI:** 10.3390/jcm12041322

**Published:** 2023-02-07

**Authors:** Chih-Yun Kuo, Hsin-Yi Tseng, Ivo Stachiv, Chon-Haw Tsai, Yi-Chun Lai, Tomas Nikolai

**Affiliations:** 1 Department of Neurology and Centre of Clinical Neuroscience, 1st Faculty of Medicine and General University Hospital in Prague, Charles University, 12108 Prague, Czech Republic; 2 Department of Psychiatry, China Medical University Hospital, Taichung 404327, Taiwan; 3 Department of Neurology, China Medical University Hospital, Taichung 404327, Taiwan; 4 Department of Functional Materials, Institute of Physics, Czech Academy of Sciences, 18200 Prague, Czech Republic; 5 College of Medicine, China Medical University, Taichung 404327, Taiwan

**Keywords:** Alzheimer’s disease, frontotemporal lobar degeneration, frontotemporal dementia, neuropsychological assessment, cognitive impairment, bvFTD, PPA, neuroimaging

## Abstract

Neuropsychological tests (NPTs), which are routinely used in clinical practice for assessment of dementia, are also considered to be essential for differential diagnosis of Alzheimer’s disease (AD) and frontotemporal lobar degeneration (FTLD), especially the behavioral variants of frontotemporal dementia (bvFTD) and primary progressive aphasia (PPA) at their initial clinical presentations. However, the heterogeneous features of these diseases, which have many overlapping signs, make differentiation between AD and FTLD highly challenging. Moreover, NPTs were primarily developed in Western countries and for native speakers of non-tonal languages. Hence, there is an ongoing dispute over the validity and reliability of these tests in culturally different and typologically diverse language populations. The purpose of this case series was to examine which of the NPTs adjusted for Taiwanese society may be used to distinguish these two diseases. Since AD and FTLD have different effects on individuals’ brain, we combined NPTs with neuroimaging. We found that participants diagnosed with FTLD had lower scores in NPTs assessing language or social cognition than AD participants. PPA participants also had lower measures in the Free and Cued Selective Reminding Test than those diagnosed with bvFTD, while bvFTD participants showed poorer performances in the behavioral measures than PPA participants. In addition, the initial diagnosis was supported by the standard one-year clinical follow-up.

## 1. Introduction

Alzheimer’s disease (AD) is a leading cause of dementia that accounts for more than 60% of all dementia cases [1]. AD is characterized by an aggregation of the extracellular *β*-amyloid plaques and intraneuronal neurofibrillary (tau) tangles in the brain [2,3]. Typical symptoms of AD that, in time, significantly affect the daily-life functioning of individuals include memory impairment, executive and language functioning problems, and changes in individuals’ behavior, such as the development of anxiety or depression [4,5]. During the past two decades, the enormous progress in understanding the underlying mechanisms of AD has made it possible to (i) propose multiple strategies for disease treatment, such as preventive single- and multidomain interventions [6,7]; and (ii) develop “possibly” disease-modifying drugs [8]. The majority of these new therapeutics are expected to target early-stage AD [9]. Thus, prospective success in the treatment of AD would also require reliable methods for the accurate clinical diagnosis of this brain disease at its earliest stage [10]. However, there is still an intense debate about the efficiency of these newly developed disease-modifying drugs [11,12].

AD pathology begins many years prior to the appearance of the first clinical symptoms. Unfortunately, the clinical diagnosis of the earliest (i.e., preclinical) stage of AD, which is characterized by the absence of clinical symptoms, is still only at the research phase [13]. Mild cognitive impairment (MCI)—in particular, amnestic MCI (aMCI)—due to AD can be considered the prodrome of dementia [14] and, correspondingly, it may be the earliest clinical stage of the disease that can be diagnosed by physicians [10]. Importantly, this stage of AD (hereafter referred to as MCI/AD) can sometimes be observed in individuals younger than 65 years of age [14]; therefore, it can easily be misdiagnosed as other types of early-onset dementia, especially frontotemporal lobar degeneration (FTLD) [15]. It should be noted that the most common types of FTLD are the behavioral variant of FTLD (bvFTD) and primary progressive aphasia (PPA). Specifically, bvFTD accounts for nearly 60% of FTLD cases, while PPA is less common, with a prevalence of almost 40% of FTLD cases [16]. Individuals diagnosed with bvFTD may often evidence strong personality and behavioral changes, such as apathy or compulsive behavior, whereas those with PPA usually show a decline in speech and language abilities [16]. As a result, for individuals younger than 65 years of age, the many overlapping signs and symptoms of MCI/AD and FTLD, such as memory impairment, language problems, and changes in individuals’ behavior, can make accurate differential diagnosis between MCI/AD, bvFTD, and PPA at their initial clinical presentations challenging. Consequently, such diagnoses are important for both research and clinical practice [17,18,19].

Numerous studies have already been performed to (i) investigate the overlapping signs of MCI/AD and FTLD and (ii) evaluate whether the standard neuropsychological tests (NPTs) may help to distinguish these two diseases [19,20,21]. Despite the enormous efforts to assess the capabilities of standard NPTs to guide the differential diagnosis of MCI/AD and FTLD, the diagnostic accuracy of NPTs may often be insufficient [22,23]. Hence, researchers and clinicians are still discussing which of the standard NPTs can be employed in clinical practice for an accurate differential diagnosis of MCI/AD and FTLD [19,24,25]. Surprisingly, some studies even provide evidence suggesting that the current clinical criteria are not sufficient when addressing early-stage FTLD (i.e., the clinical scales and clinical criteria assessment for FTLD are not standardized, and many of its early symptoms can easily overlap with other subtypes of dementia) [21,26]. Furthermore, the standard NPTs were originally developed in homogeneous English-speaking societies (i.e., for non-tonal native language speakers); therefore, some of these tests and normative data may not be suitable for culturally different and typologically diverse language populations [27,28]. The importance of these culture and language differences for diagnostic criteria was already recognized a decade ago [29]. Briefly, using appropriate constructs in questions within the neuropsychological tests—in particular, those assessing language and social cognition—is important for correct diagnosis (i.e., construct irrelevance and inappropriate test measures or incorrectly administrating neuropsychological tests may easily result in misdiagnosis) [30].

Up-to-date data on MCI/AD and FTLD from non-Western countries—especially East Asian countries, such as China, Korea, Japan or Taiwan—are still underrepresented in the scientific literature and, correspondingly, insufficient to assess the general validity of the standard NPTs in differential diagnosis of early-stage AD (MCI/AD) and FTLD (bvFTD/PPA) [31,32,33]. For example, one of the earliest studies focusing on the reliability and sensitivity of the Chinese variant of the Montreal Cognitive Assessment (MoCA-C), the Mini-Mental State Examination (MMSE), the Clinical Dementia Rating (CDR), the Audio Verbal Learning Test, and the Instrumental Activities of Daily Living Scale in the diagnosis of aMCI in Chinese populations was performed only a decade ago by Zhao et al. [34]. They showed that, for Chinese people (i.e., those living in mainland China), the MoCA-C test may help to accurately distinguish MCI individuals from normal ones, while the reliabilities determined by other studies considering NPTs in diagnoses of MCI were not confirmed. Interestingly, MoCA-C scores were much lower than those observed for Western individuals but similar to those reported in a similar study performed in Korea [35]. The reliability of MoCA-C for the determination of MCI in the Chinese population was later also confirmed by Chen et al. [36]. These pioneer studies indicated that some NPTs (particularly those assessing language and/or social cognition) have to be adjusted to reflect culture and language differences between Western and East Asian societies. Surprisingly, the capability of the Chinese version of the Social Cognition and Emotional Assessment (SEA) (a combination of the theory of mind test (ToMT) and social recognition) to discriminate MCI/AD from bvFTD in Chinese individuals living in mainland China has only recently been examined [37]. We emphasize that early-stage FTLD (i.e., bvFTD and PPA) is difficult to accurately diagnose because the necessary definite biomarkers are still not available [38]. Nevertheless, it is expected that, with the recent progress in biosensors, the necessary biomarkers should be identified and detected [39].

In clinical practice, magnetic resonance imaging (MRI), single-photon emission computed tomography (SPECT), and positron emission tomography (PET) can be used to assist in the differential diagnosis of FTLD (i.e., bvFTD and PPA) and MCI/AD (i.e., a possible prodromal stage of AD) [40]. We remind the reader that brain scans of individuals with AD usually show brain atrophy, tissue abnormalities, and hypoperfusion. Moreover, PET characterization of the *β*-amyloid and tau tangles in the brain is often considered to be essential to support the clinical diagnosis of MCI/AD [41]. On the other hand, the main limitations of PET are the prolonged acquisition time (usually more than 30 min), its restricted use in patients with metal implants or medical devices in the body, and the rarely shown pathognomonic changes in the MCI stage [42,43].

To the best of our knowledge, no other studies on differential diagnosis between MCI/AD and FTLD have been performed in Taiwan. However, many researchers expect that the main findings of studies performed in China may be applied to Taiwanese society. It is noteworthy that there are some serious differences between Taiwanese and Chinese societies, which should be reflected in NPTs; in particular, in those assessing language and social cognition (e.g., for some individuals, the “Taiwanese dialect” may be considered as their first language, and the Chinese language is then their second language, etc.) [44]. It also remains unclear whether the NPTs that have been adjusted to Taiwanese society and used in clinical practice in Taiwan may help to distinguish these two diseases. Thus, the objective of this case series was to employ these standard NPTs at the initial clinical presentation to distinguish MCI/AD from FTLD (i.e., bvFTD and PPA) among non-Western tonal native language-speaking individuals living in Taiwan. For example, the ToMT is usually considered suitable for early-stage diagnosis of bvFTD in Western individuals [45,46] and, as also discussed previously, when this test is adjusted to reflect culture and language differences, it can even be used for Chinese individuals living in China [37]. Similarly, it can be expected that the ToMT adjusted to Taiwanese society might also be suitable for Chinese language-speaking individuals living in Taiwan.

In our study, we employed the ToMT and other tests, such as the Free and Cued Selective Reminding Test (FCSRT), the Verbal Fluency Test (VTF), the Cognitive Abilities Screening Instruments (CASI), and the Montreal Cognitive Assessment (MoCA), adapted to fit Taiwanese society, together with other standardized NPTs (we only considered tests commonly used in clinical practice in Taiwan), such as the Mini-Mental State Examination (MMSE) and neuroimaging, with eight native Chinese language-speaking individuals living in Taiwan who were suspected of having MCI/AD, bvFTD, or PPA (in our study, all PPA individuals were diagnosed with nonfluent agrammatic PPA (nfaPPA)) and a normal control to obtain their cognitive profiles. Then, the early-stage clinical diagnosis and disease progression were supported by neuroimaging (i.e., the evidence of brain atrophy and biomarkers relevant to the fundamental pathological processes) and the standard one-year clinical follow-up.

## 2. Materials and Methods

### 2.1. Participants

**Figure 1:**
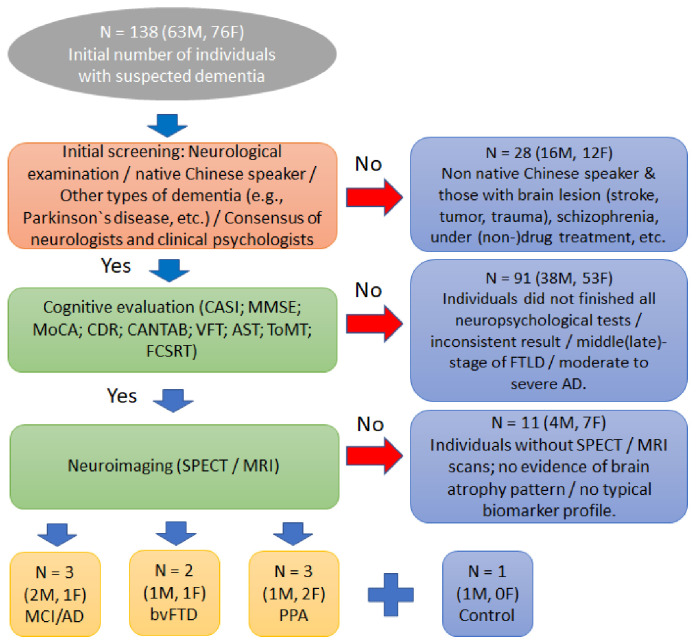
Flowchart showing the participant inclusion and exclusion criteria used in the present study. CASI—the Cognitive Abilities Screening Instruments; MMSE—the Mini-Mental State Examination; MoCA—the Montreal Cognitive Assessment; CDR—the Clinical Dementia Rating; CANTAB—the Cambridge Neuropsychological Test Automated Battery; VFT—the Verbal Fluency Test; AST—the Apraxia Screening Test; ToMT—the theory of mind test; FCSRT—the Free and Cued Selective Reminding Test; FTLD—the frontotemporal lobar degeneration; AD—Alzheimer’s disease; SPECT—single-photon emission computed tomography; MRI –magnetic resonance imaging; MCI/AD—Early stage of Alzheimer’s disease; bvFTD—behavioral variants of frontotemporal dementia; PPA—primary progressive aphasia.

The participant selection and exclusion process used in the present study is summarized in Figure 1. We initially recruited 138 participants suspected of having MCI/AD or FTLD from the Department of Neurology of the China Medical University Hospital (Taichung, Taiwan). The participant selection was based on the following inclusion criteria: (i) brain scans showing evidence of FTLD (i.e., bvFTD or PPA) or AD (i.e., evidence of appropriate biomarkers and/or brain atrophy) [47,48,49]; and (ii) individuals fulfilled the criteria for possible diagnosis of either aMCI [50] or FTLD [51]. Then, 119 participants were excluded for at least one of the following reasons: (i) brain lesion caused by stroke, tumor, or trauma detected; (ii) bipolar disorders or schizophrenia diagnosed; (iii) participant was under medical treatment (i.e., drug and/or non-drug treatments, such as drugs for memory and cognition, treatment for changes in sleep patterns or behavior, etc.); (iv) participant was a non-native speaker of the Chinese language; and (v) middle/late-stage FTLD or moderate to severe AD was diagnosed. Participants who did not finish all of the study’s designed comprehensive neuropsychological test battery and those without brain scan evaluations were also excluded. Finally, eight patients suspected of having MCI/AD or FTLD were selected. In addition, their NPT results were compared to one normal control (i.e., no symptoms or cognitive complaints, “normal” test results, and brain scans did not show any typical signs of AD or FTLD). All nine participants had also completed more than 6 years of education (the mean education level was 12.4 years (SD 3.59)), as shown in Table 1, and their final diagnoses elicited complete agreement between the neurologists and clinical psychologists. All participants (i.e., the eight patients and one control) returned to the clinic every 3 months for the standard clinical follow-up. Moreover, 12 months after their initial clinical presentations, they again completed all the present study’s designed NPTs. This study was approved by the ethic committee of the China Medical University Hospital under no. CMUH107-REC3-107.

### 2.2. Neuropsychological Tests

NPTs make it possible to assess an individual’s cognition, such as their executive functions, memory, attention, language, perception, and emotional performance. For participants suspected of having AD, a mild stage of the disease was assumed only when the scores of the Chinese variant of the MoCA and MMSE were above 18 and 20, respectively [52,53]. Similarly, for participants suspected of having FTLD, the Frontal Behavioral Inventory [54], caregiver reports, and the language ability test [55,56] were used to assess the disease progression. According to Kertesz et al. [54], scores between 25 and 30 indicate a mild to moderate stage of FTLD. The other NPTs employed in the present study for the cognitive evaluation were as follows: (i) the Chinese version of CASI [57], with a cutoff score of 79/80 (maximum score: 100); (ii) the Clinical Dementia Rating (CDR) [58], with a sum of box scores between 0.5 and 4 (i.e., scores from 0.5 to 2.5 represented questionable impairment results); (iii) the Cambridge Neuropsychological Test Automated Battery (CANTAB) [59], of which the Motor Screening Task (MOT), Rapid Visual Information Processing (RVP), Paired Associates Learning (PAL), Intra/Extra Dimensional Set Shift (IED), and Big/Little Circle (BLC) were the subtests used in the present study, with a cutoff *z* score ≤ −1.5; (iv) the Taiwanese version of the Verbal Fluency Test (VFT) [60] (the original version of the VFT can be found in the study by Zarino et al. [61]), with cutoff scores of 22 for age < 60 and 24 for age > 60; (v) the Apraxia Screening Test from TULIA (AST R/L) [62], with a cutoff score of <9 (maximum score: 12); (vi) the theory of mind test (ToMT) [63], with a cutoff score of <13 (maximum score: 20); and (vii) the Chinese cultural version of the FCSRT (the original version of the FCSRT can be found in the study by Grober et al. [64]), in which the items were adjusted to fit Chinese culture (e.g., four-legged bear was changed to four-legged elephant, desk was changed to bench, etc.). The FCSRT includes the immediate free recall (FR) and total recall (TR) (the maximum score for both the FR and TR is 48); the cued efficiency (CE) (maximum score: 1); the free delayed recall (DFR) and the total delayed recall (DTR), both with a maximum score of 16; and the delayed cued efficiency (DCE), defined as the DTR-DFR, with a maximum score of 1.

In the evaluation of language abilities, we combined behavioral observations (e.g., PPA patients have obvious difficulties in “sentence construction”), instruction comprehension, short sentence repetition, and semantic and phonemic fluency [60,65]. Clinical diagnosis of the nonfluent/agrammatic variant of PPA (nfvPPA) also requires the combination of agrammatism or speech apraxia with at least two of the following symptoms: (i) impaired comprehension of complex sentences and either unaffected (ii) single-word comprehension or iii) object knowledge [66].

### 2.3. Neuroimagining

Three-dimensional (3D) brain perfusion scans provide fundamental information on brain tissue functioning [48]. They also make it possible to identify brain atrophy and cerebrospinal fluid (CSF) biomarkers [67,68]. Neuroimaging is, therefore, of great importance for the accurate differential diagnosis of AD and FTLD at their initial clinical presentations [20]. As a result, in 2011, the NIAA-AA recommended the use of (FDG-)PET for AD biomarker characterization [69]. However, the main drawbacks of standard biomarker characterization using (FDG-)PET, which also restrict the widespread use of this technique in clinical practice, are its very high cost and difficulties in applying this method with diabetic individuals (for those with poorly controlled glucose levels, it is difficult to correctly identify the hypometabolism regions of the brain) [70]. It is noteworthy that, during the past decade, significant progress in SPECT has been achieved, making it possible to notably improve its quantification and spatial resolution [67,71,72]. Importantly, voxel-based automated analysis of the whole brain with the relevant database of normal brains [73] has been proven to have comparable diagnostic accuracy as the essentially more expensive biomarker characterization using (FDG-)PET [72].

**Figure 2:**
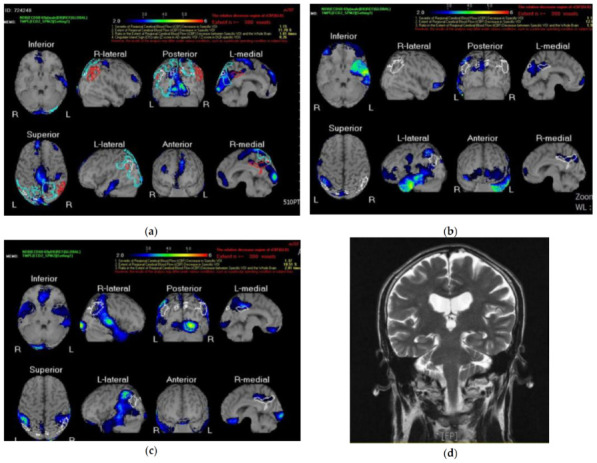
MRI/SPECT scans of the selected participants diagnosed with (**a**) bvFTD (participant no. 1), (**b**) nfvPPA (participant no. 7), (**c**) nfvPPA with atypical perfusion scan (participant no. 8), and (**d**) MCI/AD (participant no. 2). For (**a**–**c**), the perfusion SPECT images were evaluated using automated *z*-score surface mapping analysis, where the blue (red) color on the color scale represents mild (severe) disease, while for (**d**), hippocampus atrophy was determined using MRI-based hippocampal volume measurements. For *z*-score maps, the “Japanese/Chinese” gender- and age-matched database of normal brains was used to avoid age-related physiological changes in the regional cerebral blood flow.

In this study, the images of the regional changes in the cerebral blood flow that could be associated with particular dementia types and help to identify at-risk individuals with MCI were acquired with the technetium 99m ECD brain perfusion SPECT [67] and, afterwards, evaluated with a *z*-score imaging system in the Statistical Parametric Mapping analysis program [73]. These *z*-score maps of averaged SPECT images were obtained by comparing the “Japanese/Chinese” gender- and age-matched database of normal brains with SPECT images [71]. For the reader’s convenience, we show the brain perfusion SPECT data analyzed with the *z*-score surface mapping for three illustrative cases. Case 1 was a 66-year-old male with bvFTD (participant no. 1) whose brain scan is given in Figure 2a. Similarly, Figure 2b,c show the SPECT images of cases 2 and 3, which represent 62 year old (participant no. 7) and 67 year old (participant no. 8) females diagnosed with nfvPPA. Then, clinical evaluation of the brain scans for brain atrophy, tissue abnormalities, and hypoperfusion was performed by at least two independent consultants blinded to the previous neuropsychological assessments and pathological data. The visual assessments of the brain scans for hypoperfusion in each considered brain region (i.e., the right and left frontal, parietal, temporal, insula, right and left occipital, precuneus, etc.) were scored on a four-point scale from 0 to 3, where 0 indicated no hypoperfusion, 1 indicated mild hypoperfusion (i.e., the blue color on the color scale in Figure 2a,b), 2 and 3 indicated moderate hypoperfusion, and 4 indicated severe hypoperfusion (i.e., the red color on the color scale in Figure 2a,b). It should be noted that, for participants suspected of having MCI/AD, hippocampal atrophy, which can be considered an indirect biomarker for tau pathology, was determined using the standard MRI-based hippocampal volume measurement [74]. As an example, the MRI brain scan of a 65-year-old male with MCI/AD is given in Figure 2d. Finally, all brain scans were acquired in accordance with standard protocols and procedure guidelines [75]. It can be noted that that, when there is any conflict between diagnoses obtained with NPTs and MRI/SPECT, additional standard CSF biomarker profiles are used to support the correctness of the diagnoses [20]. Fortunately, in our case series, no conflicts between NPT results and neuroimaging were found.

## 3. Results

The designed neuropsychological assessment with neuroimaging was completed by nine participants (5 males and 4 females). Among them, three participants were diagnosed with MCI/AD, two with bvFTD, three with nfvPPA, and one was a control. In our study, participants with FTLD (i.e., bvFTD and nfvPPA) were younger than participants with MCI/AD at their initial clinical presentations (62.2 (SD 4.58) years old vs. 67.3 (SD 2.62) years old) and, except participant no. 4, they had lower scores in the MMSE than MCI/AD participants. Furthermore, FTLD and MCI/AD participants also had significantly different scores in the CASI, MoCA, VFT, and ToMT. Interestingly, nfvPPA participants also showed poorer performance for the VFT and FCSRT immediate and free delayed recalls, total delayed recall, and delayed cued efficiency than participants with bvFTD. The results for the CDR, AST, and CANTAB were inconsistent. For example, one patient with bvFTD (no. 1) and one with PPA (no. 8) performed poorly in the AST, while other patients with FTLD (i.e., bvFTD and nfvPPA) displayed normal performance in the AST. Scores for the key NPTs used in the present study for the cognitive evaluation are summarized for all nine participants in Table 1.

As mentioned previously, in the case of neuroimaging, a voxel-based automated analysis (the *z*-score surface mapping) of the brain perfusion SPECT, the results of which served as a clinical reference, was used [70]. For example, the brain scan of participant no. 1 (first illustrative case) showed a mild to moderate decrease in the cerebral blood flow in the left temporal lobe, bilateral frontal lobes, occipital lobe, and the posterior cingulate. At the same time, the NPT performance and outpatient observation revealed bvFTD-related symptoms, such as behavioral disinhibition, apathy, compulsive behavior, deficits in executive function, and abrupt mood changes. Participant no. 7 (second illustrative case) had a typical perfusion scan for nfvPPA (i.e., moderate to severe hypoperfusion in the left temporal lobe, a mild to moderate decrease in the cerebral blood flow in the frontal lobe and left parietal lobe, and a mild decrease in the flow in the posterior cingulate). It is worth noting that, for participant no. 8 (third illustrative case), the perfusion pattern showed a reduction in the cerebral blood flow in the bilateral frontal lobes (mild), bilateral parietal lobes (left was moderate and right was mild), bilateral temporal lobes (mild), and bilateral occipital lobes (right was moderate to severe and left was mild); therefore, it could be also interpreted as the logopedic variant of PPA [66]. In this case, the clinical diagnosis required agreement between the caregiver reports, NPT results, and outpatient observation [66]. Briefly, participant no. 8 showed altered comprehension of complex sentences (i.e., sentence construction was mostly incomplete), slurred and incoherent speech, apraxia tendencies, and difficulties in findings words. Participant no. 2 (MCI/AD), the fourth illustrative case, showed normal brain atrophy related to its ageing, mild hippocampal atrophy, and multiple hyperintensity areas in the subcortical white matter of the bilateral frontoparietal lobes (i.e., leukoaraiosis), which are usually associated with functional impairment in individuals with AD [76].

Finally, the correctness of the initial clinical diagnoses of all nine participants was supported and their disease progression assessed using the standard one-year clinical follow-up. For example, for participant no. 7 (nfvPPA), the second illustrative case, the scores for multiple NPTs were lower than those obtained at the initial clinical examination (e.g., the CASI score dropped from 68 to 56, and MMSE and VFT scores were 5 and 4 points lower than those obtained at the initial cognitive evaluation). Importantly, participant no. 8 (nfvPPA), the third illustrative case, evidenced a sharp cognitive decline within the one year before the clinical follow-up (the MMSE and CDR scores were 0 and 2, respectively). Similarly, lowered scores for the key NPTs were found for individuals diagnosed with bvFTD, while for MCI/AD participants, the observed changes in NPTs were inconsistent; this meant that no significant declines in cognitive functions were found for participants diagnosed with MCI/AD.

## 4. Discussion

This is the first study where (i) standard NPTs adjusted to Taiwanese society were used to distinguish MCI/AD from FTLD (i.e., bvFTD and nfvPPA) at the initial clinical presentation in Chinese language-speaking individuals living in Taiwan; and (ii) MRI/SPECT brain scans were primarily considered to support the initial clinical diagnosis (i.e., additional standard CSF biomarker identification [20] was only used to confirm the correctness of the MRI/SPECT). It is important to note that culturally embedded processed are reflected in neuropsychological assessments (i.e., a large number of constructs, such as language, memory, attention, and executive functioning, may be strongly culture- and language-dependent [30]). This means that some (i.e., secondary) cognitive abilities are influenced by the appropriate training (i.e., education and environment) [77]. Hence, NPTs assessing language and social cognition must be adjusted to reflect individuals’ culture and language [30,37]. As a result, the present study’s findings were primarily compared with data obtained in other Asian countries, especially mainland China.

There is a broad consensus that individuals diagnosed with aMCI are at higher risk of developing AD [14]. The clinical diagnosis of aMCI combines caregiver reports; multiple standard NPTs, such as CDR, MMSE, and MoCA; and outpatient observations [34]. Patients with aMCI can have memory problems or objective cognitive impairment (i.e., poor performance in NPTs covering episodic memory) while still being able to perform most daily life activities [50]. For example, in an early study by Zhao et al. [32], multiple NPTs, including the Chinese variant of the MoCA, were used with elderly Chinese individuals to examine whether these tests could accurately distinguish aMCI individuals from normal ones. They showed that, for individuals at high risk (i.e., those with apolipoprotein *E* carriers), these NPTs may differentiate aMCI individuals from normal ones, whereas, for low-risk individuals or when apolipoprotein *E* as a risk factor is not accounted for, these NPTs alone may not be sufficient to delineate MCI individuals from the normal Chinese population (i.e., NPT scores for MCI and normal individuals differ within the standard deviation). Our results obtained for Chinese-speaking individuals living in Taiwan were consistent with the findings of these previous studies. Specifically, all three participants with MCI/AD reported memory problems, while the scores for the NPTs did not show any significant cognitive decline (i.e., the Chinese-speaking individuals living in Taiwan had similar NPT results as those obtained previously in mainland China). In addition, their brain scan results showed evidence of leukoaraiosis and mild hippocampal atrophy, which, for MCI individuals, indicate a higher risk of developing of AD in later life [78]. It is important to note that there are also other risk factors for the development of MCI, such as stroke or depression [14,79].

The revised diagnostic criteria for bvFTD proposed by Rascovsky et al. [80] are widely used in clinical practice for assessment and diagnosis of bvFTD. These diagnostic criteria are sensitive but not yet optimal, especially when targeting earlier stages of the disease [20]. Social cognition represents the identification, perception, and interpretation of information relevant to other people and social conditions, and its impairment, such as in terms of empathy, emotional recognition, the ToMT, and social knowledge, has been observed in patients with bvFTD [81]. Since multiple tests have reported empathy impairments, a reduced ability for emotional recognition, and impaired social and executive processes [82], researchers have already suggested the use of cognitive tests targeting sociocognitive functions (e.g., the ToMT) for differential diagnosis of bvFTD and AD [45,81]. Facial expression stimuli or the Social Cognition and Emotional Assessment (SEA) are then usually employed to assess the impairment in emotional recognition [24]. As discussed previously, social cognition may be language- and culture-dependent; therefore, these tests have to be adjusted to reflect these culture differences [83,84]. For example, in the recent study by Wang et al. [35], the capability of the Chinese version of the “Mini” SEA to distinguish bvFTD from AD was examined. They showed that the Mini SEA may indeed help to differentiate bvFTD from MCI/AD. In our study, we found that participants with bvFTD had significantly lower scores in the ToMT, the Chinese version of the CASI, and the Taiwan version VFT than MCI/AD participants. In addition, bvFTD individuals had the lowest number of completed CANTAB IED stages and typical bvFTD symptoms, such as apathy, compulsive behavior, deficits in executive functioning, and abrupt mood changes [26]. Our findings are in good agreement with previously reported results on the early diagnosis of bvFTD in the Chinese population [35,85] and provide evidence supporting the use of NPTs targeting social cognition in the clinical diagnosis of bvFTD.

In contrast to bvFTD, the clinical diagnosis of PPA, including nfvPPA, in culturally different and typologically diverse language populations is still deeply understudied in the scientific literature [86]. This means that the current understanding of PPA is mainly established based on English-speaking patients [56]. The expansion of research on PPA to non-English speaking populations, especially to tonal native language-speaking individuals, requires accounting for the specificities of languages [87]. Chinese is a tonal and classifier language, where the meaning of a particular word is based on the appropriate combination of pronunciation and tone (i.e., different combinations of tone and pronunciation may have different meanings) and classifiers are necessary to quantify numeral nouns (e.g., in Chinese, when talking about a chair, one would say “one chair”) [57]. In the present study, nfvPPA participants had significantly lower scores in the VFT and FCSRT than participants diagnosed with either bvFTD or MCI/AD. Furthermore, all three participants with diagnoses of nfvPPA had clinical symptoms similar to those observed in English-speaking patients, such as problems constructing complex sentences, apraxia tendencies, and slurred and incoherent speech [88]. We emphasize here that the logopedic variant of PPA may be also due to AD [89].

Overall, our findings, together with previously published results (see, for example, [35,85,87]), provide evidence supporting the importance of adjusting NPTs to target social cognition and language (i.e., adjusted for particular societies) in the differential diagnosis of MCI/AD and FTLD (i.e., bvFTD and nfvPPA) in culturally different and typologically diverse language populations. However, the small sample size, consisting of only nine individuals (i.e., eight patients and one control), did not make it possible to undertake any statistical analysis and, correspondingly, strongly limited the interpretation of the obtained data. Other limitations of the present study are: (i) the standard PET characterization of amyloid beta and tau proteins was only undertaken to support the MRI/SPECT results (i.e., in the present study, we observed hippocampal atrophy, which may also be considered indirect evidence of tau pathology in MCI/AD); and (ii) the Taiwanese versions of the VFT and TULIA have not yet fully established local norms. Nevertheless, the obtained results are still of great importance for the design of future studies performed with larger cohorts (i.e., with non-tonal language-speaking individuals living in Taiwan or other Asian countries) in which, due to the high cost of FDG-PET (limiting the possibility of applying it to a large number of individuals), MRI and SPECT would be primarily considered to support the validity of the initial clinical diagnosis (i.e., FDG-PET could probably be used for a limited number of patients or those with an unexpected outcome).

## Figures and Tables

**Table 1:** Summarized results from the key NPTs preferably used in clinical practice in Taiwan for all participants who also completed the brain scan evaluation. PAL shows for the number of errors in six figures; IED indicates the number of completed stages of the set-shifting subtest; IED_∑_ shows the total errors and RVP designates the signal detection indicator; FR and TR are the immediate free and total recall (maximum score for both FR and TR: 48), respectively; CE (maximum score: 1) is the cued efficiency; DFR (maximum score: 16), DTR (maximum score: 16), and DCE (maximum score: 1) stand for free delayed recall, total delayed recall, and delayed cued efficiency—that is, DTR-DFR. * and bold text indicate cognitive impairment.

Participant No. (Age)/Sex/Years of Education	Type of Dementia (bvFTD; nfvPPA; MCI/AD; Control)	NPTs								
		CASI	MMSE	MoCA	CDR	VFT	AST (L/R)	ToMT	CANTAB (PAL/IED/IED_∑_/RVP)	FCSRT (FR/TR/CE/DFR/DTR/DCE)
1 (66)/M/9	bvFTD	74 *	25	18 *	1	11 *	7 */9	7 *	−4.01/3 */−5.86/0.82 *	18/48/1/13/16/1
2 (65)/M/16	MCI/AD	87	28	25 *	0.5	27	12/12	13	0.24/7 */−1.36/0.84 *	18/48/1/10/16/1
3 (71)/M/6	MCI/AD	88	27	26	0.5	43	12/12	15	0.61/8/0.02/0.72 *	31/48/1/14/16/1
4 (62)/M/12	nfvPPA	73 *	26	18 *	0.5	5 *	12/12	4 *	0.63/9/−0.43/0.91 *	10 */27/0.5/4 */8 */0.3 *
5 (54)/F/16	bvFTD	66 *	25	18 *	0.5	16 *	12/12	8 *	−5.67/2 */−4.43/0.90 *	36/48/1/12/16/1
6 (66)/F/9	MCI/AD	98	27	29	0.5	30	12/12	17	−0.86/9/−0.34/0.92	29/48/1/10/14/0.7
7 (62)/F/16	nfvPPA	68 *	23 *	16 *	0.5	8 *	12/12	9 *	−2.25/7/−0.71/0.89 *	5 */48/1/0 */6 */0.3 *
8 (67)/F/16	nfvPPA	52 *	13 *	13 *	0.5	10 *	8 */8 *	4 *	−1.18/9/0.15/0.86 *	9 */37/0.9/0 */4 */0.3 *
9 (64)/M/12	Control	96	30	26	0	34	12/12	18	0.82/9/0.82/0.95	36/48/1/11/15/0.8

CASI—the Cognitive Abilities Screening Instruments; MMSE—the Mini-Mental State Examination; MoCA—the Montreal Cognitive Assessment; CDR—the Clinical Dementia Rating; CANTAB—the Cambridge Neuropsychological Test Automated Battery; VFT—the Verbal Fluency Test; AST—the Apraxia Screening Test; ToMT—the theory of mind test; FCSRT—the Free and Cued Selective Reminding Test; FTLD—the frontotemporal lobar degeneration; AD—Alzheimer’s disease; SPECT—single-photon emission computed tomography; MRI—magnetic resonance imaging; MCI/AD—Early stage of Alzheimer’s disease; bvFTD—behavioral variants of frontotemporal dementia; nfvPPA—nonfluent nonfluent/agrammatic variant of primary progressive aphasia; and Superscript “*” stands for cognitive impairment.

## Data Availability

Not applicable.

## References

[1] Alzheimer’s Association (2021). Alzheimer’s Disease Facts and Figures.

[2] Kuo C.Y., Hsiao H.T., Lo I.H., Nikolai T. (2021). Association between obstructive sleep apnea, its treatment, and Alzheimer’s disease: Systematic mini-review. Front. Aging Neurosci..

[3] Rodriguez J.L., Karikari T., Suárez-Calvet M., Troakes C., King A., Emersic A., Aarsland D., Hye A., Zetterberg H., Blennow K. (2020). Plasma p-tau181 accurately predicts Alzheimer’s disease pathology at least 8 years prior to post-mortem and improves the clinical characterization of cognitive decline. Acta Neuropathol..

[4] Singh-Manoux A., Dugravot A., Fournier A., Abell J., Ebmeier K., Kivimaki M., Sabia S. (2017). Trajectories of depressive symptoms before diagnosis of dementia: A 28-year follow-up study. JAMA Psychiatry.

[5] Villain N., Dubois B. (2019). Alzheimer’s disease including focal presentations. Semin. Neurol..

[6] Kivipelto M., Mangialasche F., Ngandu T. (2018). Lifestyle interventions to prevent cognitive impairment, dementia and Alzheimer’s disease. Nat. Rev. Neurol..

[7] Kuo C.Y., Stachiv I., Nikolai T. (2020). Association of Late Life Depression, (Non-) Modifiable Risk and Protective Factors with Dementia and Alzheimer’s Disease: Literature Review on Current Evidences, Preventive Interventions and Possible Future Trends in Prevention and Treatment of Dementia. Int. J. Environ. Res. Public Health.

[8] Cummings J.L., Tong G., Ballard C. (2019). Treatment combinations for Alzheimer’s disease: Current and future pharmacotherapy options. J. Alzheimer’s Dis..

[9] Frozza R.L., Lourenco M.V., De Felice F.G. (2018). Challenges for Alzheimer’s disease therapy: Insight from novel mechanisms beyond memory defects. Front. Neorosci..

[10] Rasmussen J., Langerman H. (2019). Alzheimer’s disease—Why we need early diagnosis. Degener. Neurol. Neuromuscul Dis..

[11] Karran E., De Strooper B. (2022). The amyloid hypothesis in Alzheimer disease: New insights from new therapeutics. Nat. Rev. Drug Discov..

[12] Knopman D.S., Jones D.T., Greicius M.D. (2021). Failure to demonstrate efficacy of aducanumab: An analysis of the EMERGE and ENGAGE trials as reported by Biogen, December 2019. Alzheimers Dement..

[13] Di Costanzo A., Paris D., Melck D., Angiolillo A., Corso G., Maniscalco M., Motta A. (2020). Blood biomarkers indicate that the preclinical stages of Alzheimer’s disease present overlapping molecular features. Sci. Rep..

[14] Gauthier S., Reisberg B., Zaudig M., Petersen R.C., Ritchie K., Broich K., Belleville S., Brodaty H., Bennett D., Chertkow H. (2006). International Psychogeriatric Association Expert Conference on mild cognitive impairment. Mild cognitive impairment. Lancet.

[15] Draper B., Cations M., White F., Trollor J., Loy C., Brodaty H., Sachdev P., Gonski P., Demirkol A., Cumming R.G. (2016). Time of diagnosis in young-onset dementia and its determinants: The INSPIRED study. Int. J. Geriatr. Psychiatry..

[16] Onyike C.U., Diehl-Schmid J. (2013). The epidemiology of frontotemporal dementia. Int. Rev. Psychiatry.

[17] Katisko K., Cajanus A., Korhonen T., Remes A.M., Haapasalo A., Solje E. (2019). Prodromal and Early bvFTD: Evaluating Clinical Features and Current Biomarkers. Front. Neurosci..

[18] Nichols E., Szoeke C.E., Vollset S.E., Abbasi N., Abd-Allah F., Abdela J., Aichour M.T.E., Akinyemi R.O., Alahdab F., Asgedom S.W. (2019). Global, regional, and national burden of Alzheimer’s disease and other dementias, 1990–2016: S systematic analysis for the Global burden of disease study 2016. Lancet Neurol..

[19] Reul S., Lohmann H., Weindl H., Duning T., Johnen A. (2017). Can cognitive assessment really discriminate early stages of Alzheimer’s disease and behavioral variant frontotemporal dementia at initial clinical presentation?. Alzheimers Res. Ther..

[20] Krudop W.A., Dols A., Kerssens C.J., Eikelenboom P., Prins N.D., Möller C., Schouws S., Rhebergen D., van Exel E., van der Flier W.M. (2017). The pitfall of behavioral variant frontotemporal dementia mimics despite multidisciplinary application of the FTDC criteria. J. Alzheimer’s Dis..

[21] Musa G., Slachevsky A., Muñoz-Neira C., Méndez-Orellana C., Villagra R., González-Billault C., Ibáñez A., Hornberger M., Lillo P. (2020). Alzheimer’s Disease or Behavioral Variant Frontotemporal Dementia? Review of Key Points Toward an Accurate Clinical and Neuropsychological Diagnosis. J. Alzheimer’s Dis..

[22] Mendez M.F., Shapira J.S., McMurtray A., Licht E., Miller B.L. (2007). Accuracy of the Clinical Evaluation for Frontotemporal Dementia. Arch. Neurol..

[23] Leroy M., Bertoux M., Skrobala E., Mode E., Adnet-Bonte C., Le Ber I., Bombois S., Cassagnaud P., Chen Y., Deramecourt V. (2021). Characteristics and progression of patients with frontotemporal dementia in a regional memory clinic network. Alz. Res. Therapy.

[24] Ducharme S., Dols A., LaForce R., Devenney E., Kumfor F., Stock J.V.D., Dallaire-Théroux C., Seelaar H., Gossink F., Vijverberg E. (2020). Recommendations to distinguish behavioral variant frontotemporal dementia from psychiatric disorders. Brain.

[25] Ma D., Lu D., Popuri K., Wang L., Beg M.F., Alzheimer’s Disease Neuroimaging Initiative (2020). Differential Diagnosis of Frontotemporal Dementia, Alzheimer’s Disease, and Normal Aging Using a Multi-Scale Multi-Type Feature Generative Adversarial Deep Neural Network on Structural Magnetic Resonance Images. Front. Neurosci..

[26] Johnen A., Bertoux M. (2019). Psychological and cognitive markers of behavioral variant frontotemporal dementia- A clinical neuropsychologist’s view on diagnostic criteria and beyond. Front. Neurol..

[27] Ng K.P., Chiew H.J., Lim L., Rosa-Neto P., Kandiah N., Gauthier S. (2018). The influence of language and culture on cognitive assessment tools in the diagnosis of early cognitive impairment and dementia. Expert Rev. Neurother..

[28] Li H., Li C., Wang A., Qi Y., Feng W., Hou C., Tao L., Liu X., Li X., Wang W. (2020). Associations between social and intellectual activities with cognitive trajectories in Chinese middle-aged and older adults: A nationally representative cohort study. Alz. Res. Therapy.

[29] American Psychiatric Association (2013). The Diagnostic and Statistical Manual of Mental Disorders.

[30] Daugherty J.C., Puente A.E., Fasfous A.F., Hidalgo-Ruzzante N., Perez-Garcia M. (2017). Diagnostic mistakes of culturally diverse individuals when using North American neuropsychological tests. Appl. Neuropsychol. Adult..

[31] Wu Y.-T., Brayne C., Matthews F.E. (2015). Prevalence of dementia in East Asia: A systematic review of time trends. Int. J. Geriatr. Psychiatry.

[32] Kucukguc O., Soylemez B.A., Yener G., Barutcu C.D., Akyol M.A. (2017). Examining factors affecting caregiver burden: A comparison of frontotemporal dementia and Alzheimer’s disease. Am. J. Alzheimer’s Dis. Other Dement..

[33] Wong H.Y., Zhong H., Zhong M., Zhou X., Chan P.Y.C., Kwok T.C.Y., Mok K., Hardy J., Ip F.C., Fu A.K. (2022). Demographics and medication use of patients with late-onset Alzheimer’s disease in Hong Kong. J. Alzheimers Dis..

[34] Zhao S., Guo C., Wang M., Chen W., Wu Y., Tang W. (2011). A clinical memory battery for screening for mild cognitive impairment in elderly Chinese population. J. Clin. Neurosci..

[35] Lee J.-Y., Lee D.W., Cho S.-J., Na D.L., Jeon H.J., Kim S.-K., Lee Y.R., Youn J.-H., Kwon M., Lee J.-H. (2008). Brief Screening for Mild Cognitive Impairment in Elderly Outpatient Clinic: Validation of the Korean Version of the Montreal Cognitive Assessment. J. Geriatr. Psychiatry Neurol..

[36] Chen K.-L., Xu Y., Chu A.-Q., Ding D., Liang X.-N., Nasreddine Z.S., Dong Q., Hong Z., Zhao Q.-H., Guo Q.-H. (2016). Validation of the Chinese version of Montreal cognitive assessment basic for screening mild cognitive impairment. J. Am. Geriatr. Soc..

[37] Wang F., Zhou A., Wei C., Zuo X., Ma X., Zhao L., Jin H., Li Y., Guo D., Jia J. (2022). Good performance of the Chinese Version of Mini social cognition and emotional assessment in the early diagnosis of behavioral variant frontotemporal dementia. Front. Neurol..

[38] Swift I.J., Sogorb-Esteve A., Heller C., Synofzik M., Otto M., Graff C., Galimberti D., Todd E., Heslegrave A.J., van der Ende E.L. (2021). Fluid biomarkers in frontotemporal dementia: Past, present and future. J. Neurol. Neurosurg. Psychiatry.

[39] Stachiv I., Kuo C.-Y., Li W. (2023). Protein adsorption by nanomechanical mass spectrometry: Beyond the real-time molecular weighting. Front. Mol. Biosci..

[40] Albert M.S., DeKosky S.T., Dickson D., Dubois B., Feldman H.H., Fox N.C., Gamst A., Holtzman D.M., Jagust W.J., Petersen R.C. (2011). The diagnosis of mild cognitive impairment due to Alzheimer’s disease: Recommendations from the National Institute on Aging-Alzheimer’s Association workgroups on diagnostic guidelines for Alzheimer’s disease. Alzheimers Dement..

[41] Cohen A.D., Landau S.M., Snitz B.E., Klunk W.E., Blennow K., Zetterberg H. (2019). Fluid and PET biomarkers for amyloid pathology in Alzheimer’s disease. Mol. Cell. Neurosci..

[42] Albert M., DeCarli C., DeKosky S., de Leon M., Foster N.L., Fox N., Frank R., Frackowiak R., Jack C., Jagus W.J. (2005). The use of MRI and PET for clinical diagnosis of dementia and investigation of cognitive impairment: A consensus report. Alzheimer’s Assoc. Neuroimaging Work Gr. Consens. Rep..

[43] Staffaroni A.M., Elahi F.M., McDermott D., Marton K., Karageorgiou E., Sacco S. (2017). Neuroimiging in Dementia. Semin. Neurol..

[44] Qi W., Sun X., Hong Y. (2022). Normative Data for Adult Mandarin-Speaking Populations: A Systematic Review of Performance-Based Neuropsychological Instruments. J. Int. Neuropsychol. Soc..

[45] Bora E., Walterfang M., Velakoulis D. (2015). Theory of mind in behavioural-variant frontotemporal dementia and Alzheimer’s disease: A meta-analysis. J. Neurol. Neurosurg. Psychiatry.

[46] Giovagnoli A.R., Bell B., Erbetta A., Paterlini C., Bugiani O. (2019). Analyzing theory of mind impairment in patients with behavioral variant frontotemporal dementia. Neurol. Sci..

[47] Varma A.R., Adams W., Lloyd J.J., Carson K.J., Snowden J.S., Testa H.J., Jackson A., Neary D. (2002). Diagnostic patterns of regional atrophy on MRI and regional cerebral blood flow change on SPECT in young onset patients with Alzheimer’s disease, frontotemporal dementia and vascular dementia. Acta Neurol. Scand..

[48] Valotassiou V., Malamitsi J., Papatriantafyllou J., Dardiotis E., Tsougos I., Psimadis D., Alexiou S., Hadjigeorgiou G., Georgoulias P. (2018). SPECT and PET imaging in Alzheimer’s disease. Ann. Nucl. Med..

[49] Rivas-Fernández M.A., Lindín M., Zurrón M., Diaz F., Aldrey-Vázquez J.M., Pías-Peleteiro J.M., Vázquez-Vázquez L., Pereiro A.X., Lojo-Seoane C., Nieto-Vieites A. (2022). Brain Atrophy and Clinical Characterization of Adults With Mild Cognitive Impairment and Different Cerebrospinal Fluid Biomarker Profiles According to the AT(N) Research Framework of Alzheimer’s Disease. Front. Hum. Neurosci..

[50] Petersen R.C., Caracciolo B., Brayne C., Gauthier S., Jelic V., Fratiglioni L. (2014). Mild cognitive impairment: A concept in evaluation. J. Intern. Med..

[51] Chare L., Hodges J.R., Leyton C.E., McGinley C., Tan R.H., Kril J.J., Halliday G.M. (2014). New criteria for frontotemporal dementia syndromes: Clinical and pathological diagnostic implications. J. Neurol. Neurosurg. Psychiatry.

[52] Folstein M.F., Folstein S.E., McHugh P.R. (1975). “Mini-mental state”. A practical method for grading the cognitive state of patients for the clinician. J. Psychiatr. Res..

[53] Nasreddine Z.S., Phillips N.A., Bédirian V., Charbonneau S., Whitehead V., Collin I., Cummings J.L., Chertkow H. (2005). The Montreal Cognitive Assessment, MoCA: A brief screening tool for mild cognitive impairment. J. Am. Geriatr. Soc..

[54] Kertesz A., Davidson W., Fox H. (1997). Frontal Behavioral Inventory: Diagnostic criteria for frontal lobe dementia. Can. J. Neurol. Sci..

[55] Mesulam M.M. (2001). Primary progressive aphasia. Ann. Neurol..

[56] Marshall C.R., Hardy C.J.D., Volkmer A., Russell L.L., Bond R.L., Fletcher P.D., Clark C.N., Mummery C.J., Schott J.M., Rossor M.N. (2018). Primary progressive aphasia: A clinical approach. J. Neurol..

[57] Liu X., Wang W., Wang H., Sun Y. (2019). Sentence comprehension in patients with dementia of the Alzheimer’s type. PeerJ.

[58] O’Bryant S.E., Waring S.C., Cullum C.M., Hall J., Lacritz L., Massman P.J., Lupo P.J., Reisch J.S., Doody R., Texas Alzheimer’s Research Consortium (2008). Stating dementia using clinical dementia rating scale sum of boxes scores. Arch. Neurol..

[59] Sahakian B.J., Morris R.G., Evenden J.L., Heald A., Levy R., Philpot M., Robbins T.W. (1988). A Comparative Study of Visuospatial Memory and Learning in Alzheimer-Type Dementia and Parkinson’s Disease. Brain.

[60] Chung S.-Y., Hua M.-S., Hsuech H.-C., Chang Y.-S., Chiu C.-F., Chen M.-C. (2007). The Performance Pattern of Normal Illiterate and Patients with Early Alzheimer’s Disease on the Semantic Association of Verbal Fluency Test. Chin. J. Psychol..

[61] Zarino B., Crespi M., Launi M., Casarotti A. (2014). A new standardization of semantic verbal fluency test. Neurol. Sci..

[62] Johnen A., Frommever J., Modes F., Wiendl H., Duning T., Lohmann H. (2016). Dementia Apraxia Test (DATE): A Brief Tool to Differentiate Behavioral Variant Frontotemporal Dementia from Alzheimer’s Dementia Based on Apraxia Profiles. J. Alzheimer’s Dis..

[63] Gregory C., Lough S., Stone V., Erzinclioglu S., Martin L., Baron-Cohen S., Hodges J.R. (2002). Theory of mind in patients with frontal variant frontotemporal dementia and Alzheimer’s disease: Theoretical and practical implications. Brain.

[64] Grober E., Sanders A.E., Hall C., Lipton R.B. (2010). Free and Cued Selective Reminding Identifies Very Mild Dementia in Primary Care. Alzheimer Dis. Assoc. Disord..

[65] Lezak M., Howieson D., Bigler E., and Tranel D. (2012). Neuropsychological Assessment.

[66] Montembeault M., Brambati S.M., Gorno-Tempini M.L., Migliaccio R. (2018). Clinical, anatomical, and pathological features in the three variants of primary progressive Aphasia: A review. Front. Neurol..

[67] Cheng Y., Ono M., Kimura H., Ueda M., Saji H. (2012). Technetium-99m Labeled Pyridyl Benzofuran Derivatives as Single Photon Emission Computed Tomography Imaging Probes for β-Amyloid Plaques in Alzheimer’s Brains. J. Med. Chem..

[68] Jack C.R., Wiste H.J., Weigand S.D., Knopman D.S., Vemuri V.L.P., Mielke M.M., Jones D.T., Senjem M.L., Gunter J.L., Gregg B.E. (2013). Amyloid-first and neurodegeneration-first profiles characterize incident amyloid PET positivity. Neurology.

[69] McKhann G.M., Knopman D.S., Chertkow H., Hyman B.T., Jack C.R., Kawas C.H. (2011). The diagnosis of dementia due to Alzheimer’s disease: Recommendations from the National Institute on Aging-Alzheimer’s Association workgroups on diagnostic guidelines for Alzheimer’s disease. Alzheimers Dement..

[70] Valotassiou V., Angelidis G., Psimadas D., Tsougos I., Georgoulias P. (2021). In the era of FDG PET, is it time for brain perfusion SPECT to gain a place in Alzheimer’s disease imaging biomarkers?. Eur. J. Nucl. Med. Mol. Imaging.

[71] Hashimoto H., Nakanishi R., Mizumura S., Hashimoto Y., Okamura Y., Yamanaka K., Ikeda T. (2020). Prognostic value of 99mTc-ECD brain perfusion SPECT in patients with atrial fibrillation and dementia. EJNMMI Res..

[72] Davison C.M., O’Brien J.T. (2014). A comparison of FDG-PET and blood flow SPECT in the diagnosis of neurodegenerative dementias: A systematic review. Int. J. Geriatr. Psychiatry.

[73] Baker J.G., Williams A.J., Wack D.S., Miletich R.S. (2013). Correlation of Cognition and SPECT Perfusion: Easy Z Score and SPM Analysis of a Pilot Sample with Cerebral Small Vessel Disease. Dement. Gediatr.Cogn. Dis..

[74] Jack C., Petersen R.C., Xu Y.C., O’Brien P.C., Smith G., Ivnik R.J., Boeve B.F., Waring S.C., Tangalos E.G., Kokmen E. (1999). Prediction of AD with MRI-based hippocampal volume in mild cognitive impairment. Neurology.

[75] Darcourt J., Booij J., Tatsch K., Varrone A., Borght T.V., Kapucu L., Någren K., Nobili F., Walker Z., Van Laere K. (2010). EANM procedure guidelines for brain neurotransmission SPECT using 123I-labelled dopamine transporter ligands, version 2. Eur. J. Nucl. Med. Mol. Imaging.

[76] Brown W.R., Moody D.M., Thore C.R., Challa V.R. (2000). Cerebrovascular pathology in Alzheimer’s disease and leukoaraiosis. Ann. N. Y. Acad. Sci..

[77] Fernandez A.L., Abe J. (2018). Bias in cross-cultural neuropsychological testing: Problems and possible solutions. Cult. Brain.

[78] Te M., Zhao E., Xingyue Z., Qinjian S., Chuanqiang Q. (2014). Leukoaraiosis with mild cognitive impairment. Neurol. Res..

[79] Kuo C.Y., Stachiv I. (2022). Biological mechanisms and possible primary prevention of depression. World J. Psychiatry.

[80] Rascovsky K., Hodges J.R., Knopman D., Mendez M.F., Kramer J.H., Neuhaus J., Van Swieten J.C., Seelaar H., Dopper E.G.P., Onyike C.U. (2011). Sensitivity of revised diagnostic criteria for the behavioral variant of frontotemporal dementia. Brain.

[81] Duclos H., Desgranges B., Eustache F., Laisney M. (2018). Impairment of social cognition in neurological diseases. Rev. Neurol..

[82] Baez S., Manes F., Huepe D., Torralva T., Fiorentino N., Richter F., Huepe-Artigas D., Ferrari J., Montaã±Es P., Reyes P.A. (2014). Primary empathy deficits in frontotemporal dementia. Front. Aging Neurosci..

[83] Koelkebeck K., Uwatoko T., Tanaka J., Kret M.E. (2017). How culture shapes social cognition deficits in mental disorders: A review. Soc. Neurosci..

[84] Kwon J.Y., Wormley A.S., Vaarnum E.W. (2021). Changing cultures, changing brains: A framework for integrating cultural neuroscience and cultural change research. Biol. Psychol..

[85] Chao S.Z., Rosen H.J., Azor V., Ong H., Tse M.M., Lai N.B., Hou C.E., Seeley W.W., Miller B.L., Matthews B.R. (2013). Frontotemporal dementia in eight Chinese individuals. Neurocase.

[86] Weekes B.S.H. (2020). Aphasia in Alzheimer’s disease and other dementias (ADOD): Evidence from Chinese. Am. J. Alzheimer’s Dis. Other Dement..

[87] Tee B.L., Tempini M.L.G., Chen T., Lo R.Y., Wang P., Chan A.L., Chen L.L.K., Wong A., Yan C.T., Tsoh J. (2021). Another side of the coin: Primary progressive aphasia in Chinese language. Alzheimers Dement..

[88] Grube M., Bruffaerts R., Schaeverbeke J., Neyens V., De Weer A.-S., Seghers A., Bergmans B., Dries E., Griffiths T.D., Vandenberghe R. (2016). Core auditory processing deficits in primary progressive aphasia. Brain.

[89] Harris J.M., Gall C., Thompson J.C., Richardson A.M., Neary D., du Plessis D., Pal P., Mann D.M., Snowden J.S., Jones M. (2013). Classification and pathology of primary progressive aphasia. Neurology.

